# Assessed versus Perceived Risks: Innovative Communications in Agri-Food Supply Chains

**DOI:** 10.3390/foods10051001

**Published:** 2021-05-03

**Authors:** Fabio G. Santeramo, Antonio Bevilacqua, Mariangela Caroprese, Barbara Speranza, Maria Giovanna Ciliberti, Marco Tappi, Emilia Lamonaca

**Affiliations:** Department of Agriculture, Food, Natural Resources and Engineering (DAFNE), University of Foggia, 71122 Foggia, Italy; antonio.bevilacqua@unifg.it (A.B.); mariangela.caroprese@unifg.it (M.C.); Barbara.speranza@unifg.it (B.S.); maria.ciliberti@unifg.it (M.G.C.); marco.tappi@unifg.it (M.T.); emilia.lamonaca@unifg.it (E.L.)

**Keywords:** food-borne illness, food safety, hazard, information, willingness to pay

## Abstract

Food preparations, especially those based on animal products, are often accused of being responsible for the increase in food-borne infections, contributing to increased pressure on healthcare systems. The risk assessment in agri-food supply chains is of utmost importance for the food industry and for policymakers. A wrong perception of risks may alter the functioning of supply chains; thus, efforts should be devoted to communicating risks in an efficient way. We adopt a multidisciplinary approach to investigate how consumers perceive different food risks. Our analysis shows that planning effective communication strategies is very much important for efficiently informing consumers on food risks. We also comment on potential innovative ways to better organise the supply chains.

## 1. Introduction

Foodborne diseases are becoming more and more frequent; depending on the type of pathogen involved, virulence, and individual state of health, they may cause morbidity and mortality worldwide [1]. In 2010, there were about 600 million foodborne disease cases and 420,000 related deaths. Viruses, bacteria, protozoa, and toxins (among other pathogens) may be passed throughout food supply chains and transferred to the consumers. In some cases, they may cause several symptoms and syndromes such as fever, diarrhea, sepsis, haemolytic uraemic syndrome, and diseases such as central nervous system infections, enteric intoxications, and hepatocellular carcinoma, or also lead to death [2]. One may argue that, due to these concerns, a solution may be to limit industrial production and adopt much more stringent standards, even if this would limit the amount of produced food. Unfortunately, this would be a rather partial, if not unfeasible, solution, as the production of food is only partially sufficient. Differently, the solution would be to produce enough food, but guarantee that it will be safe: the well-known pillars of food security [3]. The dual challenge is to ensure food safety so that the handling, preparation, and storage of food are respectful of protocols and hygienic practices to limit foodborne diseases [4] while reaching the food security status. This would ensure that safe food meets the need for healthy diets [5]. Food safety may be guaranteed through proper personal hygiene, appropriate storage, procuring food from safe sources, and cooking it at adequate temperatures [6]. Apart from the well-known thermal treatments, there are novel approaches, such as the use of ultrasounds combined with antimicrobials, that tend to be used to monitor the development of foodborne bacteria in the food industry [7]. The level of food safety may be also improved by means of policy interventions and investments to improve the transportation and communication infrastructures [8]. On the consumers’ side, the challenge is to communicate the potential risks in an effective way to limit the risks associated with the consumption of contaminated or badly prepared foods. Unfortunately, it seems that consumers tend to underestimate food safety risks due to an optimistic bias, resulting in a misperception of specific hazards [9]. Consumers’ choices are affected by several factors [10,11,12]: biological (e.g., taste), psychological (e.g., mood), physiological (e.g., access, time), social (e.g., culture, socio-cultural position), economics (e.g., cost, price, income), environmental and health factors, origin of food or animal production systems, and welfare. In short, food-risk perception is an important part of consumers’ decision-making process and calls for a deep understanding of the mechanisms behind food communications [13,14].

Following a multidisciplinary approach, the article deepens understanding of how different sources and types of risks tend to be perceived by consumers to draw conclusions on how information may be correctly and efficiently transferred to them. In particular, the article explores the effectiveness of communication in agri-food systems and comments on potential innovative ways to better organise the supply chains.

The next section of the article reviews potential risks for selected products of the agri-food industry (e.g., dairy and meat sector, fruit and vegetable sector). Section 3 explores methods to evaluate food risks and assess the risk level associated with different combinations of hazards and food products. Section 4 briefly reviews the literature on the role played by ambiguity aversion on decisions under uncertainty with specific emphasis on the potential role it plays in consumers’ choices. Section 5 evaluates, through an experimental survey, how different sources and types of risks in selected agri-food supply chains are perceived by consumers. The last section concludes the article by providing insights on the role of innovative strategies to communicate risks in agri-food supply chains in an effective way to contribute to matching assessed with perceived food risks.

## 2. Sources and Types of Risks for Selected Produce of Food Industry

Foods based on animal derivates are common and consumers are concerned about the possibility of developing antimicrobial resistance. In 2011, the European Commission introduced a five-year action plan to face the rising threats from antimicrobial resistance and develop holistic measures to limit the use of antimicrobials, in particular in dairy animals. Animal-based foods are often accused of being responsible for the increase in food-borne infections [15]. *Salmonella* spp. and *Escherichia coli* are pathogens most frequently detected in food-borne outbreaks [16,17], as shown in Table 1. In 2017, the European Union reported 91,662 confirmed human cases of salmonellosis and 6073 *Escherichia coli* infections produced by the Shiga toxin (STEC): 19.7 infections per 100,000 individuals were notified for salmonellosis and 1.66 per 100,000 individuals for STEC infection [18].

Eggs are a main ingredient in several food products: about 70% of complex foods including eggs as an ingredient are associated with illness [19]. Eggs and egg products account for 36.8% of total salmonellosis, bakery products for 16.7%, and meat and meat products for 8.2% of total salmonellosis [18]. Red meat contributes heavily to deaths associated with food-borne infections, despite lower levels of risk [15]. Outbreaks caused by STEC infection are mostly related to meat and its derivates, in particular, bovine meat and products account for 44.4%, other meat and related products for 11.1%, and milk for 22.2% of total STEC infection [18].

Food-borne infections are also caused by vegetable-based food products. *Listeria monocytogenes* and *Clostridium botulinum* are other sources of food-borne disease. They are related to the strongest case fatality among food-borne diseases, i.e., 7.7% for *Clostridium botulinum* and 5.1% for *Listeria monocytogenes* [18]. In 2017, the EU reported 2480 cases of listeriosis. The rate was 0.48 per 100,000 individuals and the case fatality was 13.8%. Fruit, vegetables, and their derivates account for 28.6% of total listeriosis disease [20]. Cases of botulism in the EU are approximately 200 per year (0.03 cases per 100,000 individuals) [21]. Primary causes of botulism are home preservation, traditional preservation, or failure of a commercial process [22].

## 3. Evaluation of Food Risks

**Figure 1:**
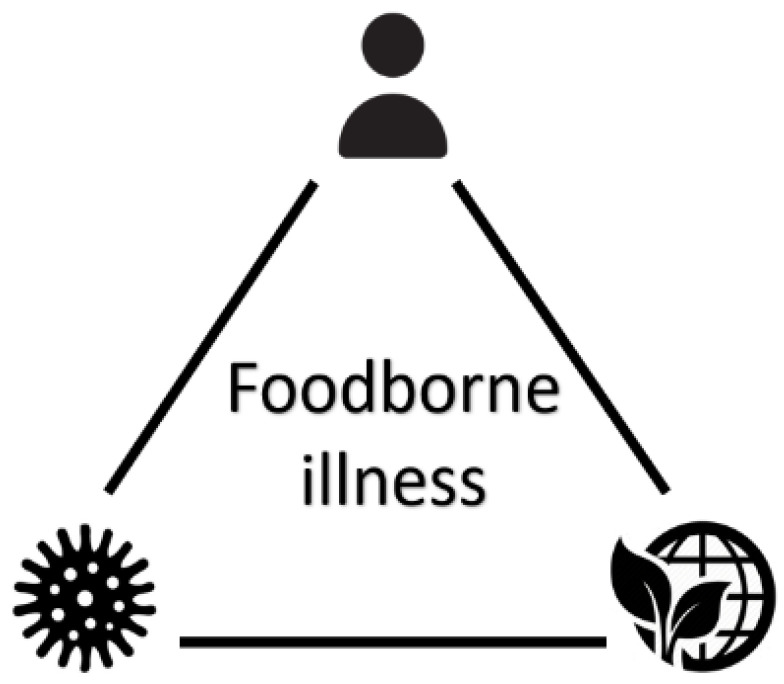
The epidemiology triangle. Source: Authors’ elaboration on Coleman and Marks [23]. Notes: The triangle reports the pathogen in the leftward angle, the host in the upward angle, the environment in the rightward angle.

According to Coleman and Marks [23], the probability and severity of diseases are highly related to characteristics of three factors and their interactions: the pathogen, such as species, virulence, dose, and growth potential in food (leftward angle in Figure 1); the host, such as health state and age (upward angle in Figure 1); and the environment, such as type of food vehicle and microbial competitors (rightward angle in Figure 1).

Several tools allow assessment of whether a pathogen may be a source of hazard for a certain food in a specific food process. Differently from semi-quantitative scoring systems and decision trees [24,25,26], stepwise approaches [27], and schemes for qualitative risk assessments [28,29], the Risk Ranger software allows identification of phases where control measures could be effectively implemented. Described in Ross and Sumner [30], the Risk Ranger software is a calculation tool based on 11 questions that allow attribution of a rank to a specific combination of food and hazard. The questions are related to the three main determinants of risk from a pathogen or toxin in a particular food product, i.e., the severity of the hazard, the dose of the hazard in a food causing the hazard, and the exposure to the hazard in a period.

The severity of the hazard depends on characteristics of the pathogen or toxin and on consumers’ susceptibility: these aspects are analysed through questions 1 and 2. Questions 3–5 allow evaluation of the absolute risk (i.e., the dose of the hazard in a food causing the hazard) as a function of the consumption frequency, the proportion of individuals consuming the product, and the size of the population of interest. The exposure to the hazard is assessed considering aspects related to the contamination of food in questions 6–9 and question 11 and the concentration of the hazard in question 10.

Qualitative information collected from the 11 questions are converted into numerical values and combined with quantitative inputs to generate indices of risk ranging between 0 (i.e., no risk) and 100 (i.e., all products contain a lethal dose of the hazard).

We used the Risk Ranger software to generate a Risk Ranking (RR) for 5 combinations of hazard and product (Table 2).

The hazard/product combinations analysed include *Clostridium botulinum* in canned food (RR = 79), *Escherichia coli* or *Salmonella* spp. in undercooked meat (RR = 59), *Listeria monocytogenes* in ready-to-eat vegetables (RR = 72), *Salmonella* spp. in undercooked eggs (RR = 50), and *Salmonella* spp. in cooked eggs (RR = 46). Based on their ranking, those hazard/product pairs fall into a high-risk category (>48).

As part of semi-quantitative methods, the risk matrix (Table 3) combines categorical labels (e.g., likelihood and severity) as possible semi-quantitative risk characterisations to determine the appropriate categorisation of the risk [31].

## 4. Decisions under Uncertainty: Ambiguous and Innovative Communication Strategies

Decisions under uncertainty are highly related to subjective determinants and characteristics of consumers and to the type of information they receive [32]. Communication in agri-food supply chains tends to be characterised by ambiguity: consumers have different capabilities in processing information (i.e., subjective factors) and are only partially informed on potential risks associated with certain foods as compared to producers and marketers (i.e., type of information). Ambiguity affects consumers’ behaviours [33]. The way consumers make choices under uncertainty is affected by channels through which information is conveyed and by processes associated with the elaboration of information received. Indeed, communication strategies about risks in agri-food supply chains may influence consumers’ attitudes and behaviours towards risky decisions [14].

**Figure 2:**
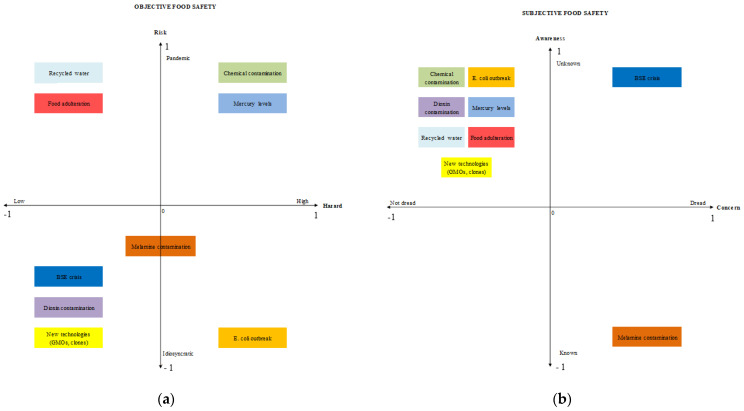
Conceptual framework elaborated in Santeramo and Lamonaca [15] to classify food-born factors. Panel (**a**) considers the objective dimensions of food safety. Panel (**b**) considers the subjective dimensions of food safety. Source: Santeramo and Lamonaca [15].

A recent paper by Santeramo and Lamonaca [15] evaluated how food risks are perceived to emphasise how food-safety information may be efficiently communicated to consumers. According to Grunert [34], there is a mismatch between assessed risks and perceived risks in agri-food supply chains. Food risks may be assessed considering two objective and scientific dimensions: the severity of the hazard and the likelihood of risk occurrence [35]. However, food risks may be differently perceived by consumers depending on their degree of aversion towards hazards and risks. The degree of aversion may depend on awareness of a certain food risk that may be known or unknown and on concern related to potential adverse effects of consuming unsafe food that counterpose not-dreadful and dreadful food-borne risks [36]. Based on these premises, Santeramo and Lamonaca [15] elaborated a conceptual framework and classify food-borne risk factors analysed in literature according to objective (i.e., assessed risks) and subjective (i.e., perceived risks) dimensions (Figure 2).

As evident from Figure 2, there is a frequent inconsistency between assessed and perceived risks in agri-food systems. The discrepancy mostly occurs in the case of food scares. A few examples are mad cow disease (BSE crisis) or dioxin contamination. Divergences between assessed and perceived food risks are also common for new technologies (e.g., GMOs, clones).

The meta-analysis by Santeramo and Lamonaca [15] also concluded on the role that communication strategies may have in reducing the gap between assessed and perceived food risks. The meta-regression results reveal that products conveying food-safety information through labels benefit from a price premium of the magnitude of +169%. It should be not neglected that, if exposed to relevant food risk information, risk perception tends to be alienated from assessed risk and decision-makers tend to reduce premium prices [32,37]. To overcome this problem, producers and marketers in agri-food supply chains should consider innovative strategies to improve the communication of food risks, avoiding losing premium prices for food safety information. Examples are innovative labels conveying information on food safety, such as traffic lights labels and nutri-score labels, or the use of nanotechnologies [38].

## 5. Consumers’ Perception of Sources and Types of Risks

An experimental survey has been developed to evaluate how consumers perceive food risks and emphasise how food safety information may be effectively communicated to consumers. Based on the review of sources and types of risks and on evidence from the Risk Ranger analysis, the following food-safety risks posed by specific hazard/product combinations have been considered:

1. botulism from *Clostridium botulinum* in canned food;
2. haemolytic uremic syndrome from *Escherichia coli* in undercooked meat;
3. listeriosis from *Listeria monocytogenes* in ready-to-eat vegetables;
4. salmonellosis from *Salmonella* spp. in under-cooked eggs.

The experimental design involved three information treatments synthesised in Table 4. In the first stage, respondents received only general information to establish a baseline level of understanding about each hazard/product combination. In the second stage, respondents received partial information linking each hazard to a food-borne disease. In the third stage, we exposed respondents to a negative and a positive message (complete information). While the negative message informed consumers on symptomatology associated with each food-borne disease, the positive message communicated that consumers may significantly reduce the risk of a food-borne infection by adopting specific prevention measures.

**Figure 3:**
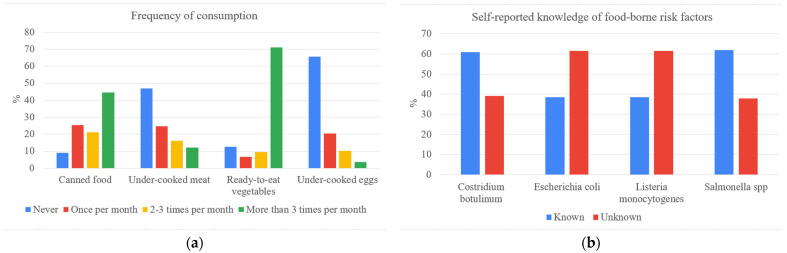
Panel (**a**) reports the frequency of consumption of food products. Panel (**b**) shows the self-reported knowledge of food-borne risk factors.

The questionnaire, preliminarily tested among selected respondents, was available from July to December 2020 as a Google Form and shared via social networks (e.g., Facebook, LinkedIn, Twitter) and e-mail lists (e.g., professional associations, producers’ groups, consumers groups). Adopting a snowball sampling recruitment allowed us to take advantage of interpersonal relations and connections among respondents. The sample consists of 166 young Italian consumers (18–35 years old). Figure 3 shows the self-reported knowledge of food-borne risk factors and the frequency of consumption of selected food products.

Respondents frequently consume ready-to-eat vegetables (more than 3 times per month) and canned food (2–3 times per month or more); under-cooked meat and eggs are consumed less. *Escherichia coli* and *Listeria monocytogenes* are known to 61% of respondents, whereas *Clostridium botulinum* and *Salmonella* spp. are known only to 39% and 38% of respondents, respectively.

In each stage (i.e., information treatments), respondents were asked to assess the risk of consuming selected foods and to specify the premium price they are willing to pay for microbiologically tested food. The results (Table 5) reveal that, moving from general to partial information, the perceived risk is unchanged for each hazard/product combination. With partial information, the match between perceived risk and assessed risk (see Table 2) occurs only for *Escherichia coli* in undercooked meat and *Salmonella* spp. in under-cooked eggs. Moving from partial to complete information, the perception of risks associated with the combinations *Clostridium botulinum*/canned food and *Listeria monocytogenes*/ready-to-eat vegetables increases but does not correspond to assessed risks.

Moving from partial to general information, the willingness-to-pay (WTP) for microbiologically tested products is unchanged. The only exception is the combination *Clostridium botulinum*/canned food with a one-percent increase in WTP (from 7% to 8%) (Table 6).

Control questions showed that only a low percentage of respondents correctly processed information provided in each treatment. This evidence highlights the need to revise the experimental design to correctly convey information to respondents.

## 6. Concluding Remarks

We examined the role of communication in food supply chains and how it may contribute to matching assessed with perceived risks and avoiding biases due to ambiguity aversion in consumers’ choices. In particular, the review of different sources and types of risks for selected food industries highlighted the incidence of food-borne infections in animal-based foods, such as meat (especially undercooked meat) and eggs and egg products (especially under-cooked eggs), as well as in ready-to-eat vegetables and canned food (especially homemade preparations). In addition, the approach used to predict food risks (i.e., Risk Ranger software) allowed identification of the assessed risk (RR) in selected hazard/product combinations, i.e., *Clostridium botulinum* in canned food (RR = 79), *Escherichia coli* in undercooked meat (RR = 59), *Listeria monocytogenes* in ready-to-eat vegetables (RR = 72), and *Salmonella* spp. in under-cooked eggs (RR = 56). Further, the role the reviewed literature played in assessing ambiguity aversion on decisions made under uncertainty revealed that, if consumers receive information on potential risks associated with the consumption of unsafe food, their risk perception increases, and their willingness to pay premium prices for information on food safety decreases.

Based on this evidence, an experimental survey aimed at evaluating how different sources and types of risks are perceived by consumers emphasised that communication in food supply chains plays a relevant role. However, the information on assessed risk is efficiently transferred to consumers only for specific hazard/product combinations, i.e., *Escherichia coli* in undercooked meat and *Salmonella* spp. in under-cooked eggs.

Implications for the food industry and policymakers are derived. Food safety in agri-food supply chains is frequently characterised by asymmetric information. Producers and marketers tend to be better informed than consumers on the potential risks of foods. Particularly relevant is the gap between assessed and perceived risks related to *Clostridium botulinum* in canned food and *Listeria monocytogenes* in ready-to-eat vegetables. Using innovative strategies to communicate information on food risks may contribute to lowering the divergence between assessed and perceived risks. In this regard, innovative labels, such as traffic lights labels and nutri-score labels, or the use of nanotechnologies may be a valid alternative. Furthermore, technologies such as Agri-Food 4.0, Blockchain, and Internet of Things may be useful tools to inform consumers in real-time, also supporting the supply chain decision-making process [43] and improving the coordination process that involves farmers, industries, and consumers [44].

Nonetheless, correctly conveying the information is challenging and needs to be further investigated. This emerges also from the results of the experimental survey (i.e., only a low percentage of respondents correctly processed information). To overcome this limitation, further research is needed to reorganise the structure of the experimental design to provide further types of information. Further research in this direction would provide a clearer understanding of how consumers assess food-safety risks and how much they are willing to pay for labels indicating safe food.

## Figures and Tables

**Table 1:** Reported cases due to zoonoses in the European Union, 2017.

Disease	Confirmed Cases	Hospitalised Cases	Case Fatality (%)	Cases per 100,000 Individuals (%)
Salmonellosis	91,662	16,796	0.25	19.70
STEC infection	6073	933	0.50	1.66

Source: Elaboration on EFSA and ECDC [18]. Notes: STEC stands for Shiga toxin-producing *Escherichia coli*.

**Table 2:** Risk assessment from target food-borne risk factors in selected carriers.

Food-Borne Risk Factor	*Clostridium botulinum*	*Escherichia coli/* *Salmonella* spp.	*Listeria monocytogenes*	*Salmonella* spp.	
Carrier	Canned Food	Undercooked Meat	Ready-to-Eat Vegetables	Undercooked Eggs	Cooked Eggs
Q1: Hazard severity	severe	moderate	moderate	mild	mild
Q2: Consumer’s susceptibility	general	general	general	general	general
Q3: Consumption frequency	monthly	weekly	weekly	weekly	weekly
Q4: Individuals consuming	most	most	most	some	most
Q5: Population size					
Q6: Proportion of contaminated food	infrequent	sometimes	sometimes	sometimes	sometimes
Q7: Process effect on contamination	usually eliminates	slightly	no effect	slightly	usually
Q8: Potential recontamination	no	minor	minor	minor	minor
Q9: Level of hazard throughout the supply chain	not relevant	not controlled	not controlled	not controlled	not controlled
Q10: Effectiveness of the post-processing control system	none	significant	moderate	significant	significant
Q11: Preparation effect on contamination	no effect	no effect	no effect	no effect	no effect
Risk	79	59	72	50	46

Source: Authors’ elaboration based on Risk Ranger.

**Table 3:** Risk matrix.

Labels	Negligible	Minor	Moderate	Significant	Severe
Very likely	**	***	****	*****	*****
Likely	*	**	***	****	*****
Possible	*	**	***	****	****
Unlikely	*	**	**	***	****

Notes: The risk matrix combines the likelihood and severity of risks at different value scales that correspond to low risk (*), low-middle risk (**), middle risk (***), middle-high risk (****), and high risk (*****).

**Table 4:** Information treatments.

Food-Borne Risk Factor	General Information	Partial Information	Complete Information
*Clostridium botulinum*	May be present in canned food	May cause botulism	Symptoms and prevention measures
*Escherichia coli*	May be present in undercooked meat	May cause haemolytic uremic syndrome	
*Listeria monocytogenes*	May be present in ready-to-eat vegetables	May cause listeriosis	
*Salmonella* spp.	May be present in undercooked eggs	May cause salmonellosis	

Source: elaboration on information from EpiCentro 2020 [39,40,41,42].

**Table 5:** Assessed versus perceived risks.

Food-Borne Risk Factor	Product	Assessed Risk	Perceived Risk		
			General Information	Partial Information	Complete Information
*Clostridium botulinum*	Canned food	79%	20–40%	20–40%	40–60%
*Escherichia coli*	Under-cooked meat	59%	40–60%	40–60%	40–60%
*Listeria monocytogenes*	Ready-to-eat vegetables	72%	40%	40%	40–60%
*Salmonella* spp.	Under-cooked eggs	56%	40–60%	40–60%	40–60%

Notes: average values for subjective risks.

**Table 6:** Willingness to pay for microbiologically tested products.

Food-Borne Risk Factor	Product	Partial Information	Complete Information
*Clostridium botulinum*	Canned food	7%	8%
*Escherichia coli*	Under-cooked meat	8%	8%
*Listeria monocytogenes*	Ready-to-eat vegetables	8%	8%
*Salmonella* spp.	Under-cooked eggs	8%	8%

## Data Availability

Not applicable.
